# A physiological signature of sound meaning in dementia

**DOI:** 10.1016/j.cortex.2016.01.007

**Published:** 2016-04

**Authors:** Phillip D. Fletcher, Jennifer M. Nicholas, Laura E. Downey, Hannah L. Golden, Camilla N. Clark, Carolina Pires, Jennifer L. Agustus, Catherine J. Mummery, Jonathan M. Schott, Jonathan D. Rohrer, Sebastian J. Crutch, Jason D. Warren

**Affiliations:** a Dementia Research Centre, UCL Institute of Neurology, University College London, London, United Kingdom; b London School of Hygiene and Tropical Medicine, University of London, London, United Kingdom

**Keywords:** Nonverbal sound, Semantic, Pupillometry, Physiology, Dementia, Alzheimer's disease, Frontotemporal, Progressive aphasia

## Abstract

The meaning of sensory objects is often behaviourally and biologically salient and decoding of semantic salience is potentially vulnerable in dementia. However, it remains unclear how sensory semantic processing is linked to physiological mechanisms for coding object salience and how that linkage is affected by neurodegenerative diseases. Here we addressed this issue using the paradigm of complex sounds. We used pupillometry to compare physiological responses to real versus synthetic nonverbal sounds in patients with canonical dementia syndromes (behavioural variant frontotemporal dementia – bvFTD, semantic dementia – SD; progressive nonfluent aphasia – PNFA; typical Alzheimer's disease – AD) relative to healthy older individuals. Nonverbal auditory semantic competence was assessed using a novel within-modality sound classification task and neuroanatomical associations of pupillary responses were assessed using voxel-based morphometry (VBM) of patients' brain MR images. After taking affective stimulus factors into account, patients with SD and AD showed significantly increased pupil responses to real versus synthetic sounds relative to healthy controls. The bvFTD, SD and AD groups had a nonverbal auditory semantic deficit relative to healthy controls and nonverbal auditory semantic performance was inversely correlated with the magnitude of the enhanced pupil response to real versus synthetic sounds across the patient cohort. A region of interest analysis demonstrated neuroanatomical associations of overall pupil reactivity and differential pupil reactivity to sound semantic content in superior colliculus and left anterior temporal cortex respectively. Our findings suggest that autonomic coding of auditory semantic ambiguity in the setting of a damaged semantic system may constitute a novel physiological signature of neurodegenerative diseases.

## 1 Introduction

Disambiguation of potentially relevant, ‘salient’ stimuli from the busy multisensory background is accomplished efficiently and largely automatically by the healthy brain. However, successful processing of sensory salience depends on a number of subprocesses: these include accurate parsing of the sensory environment, representation of particular sensory objects, assignment of emotional and reward value, and linkage to physiological and motor effector mechanisms that govern an appropriate behavioural response (Beissner et al., 2013, Critchley et al., 2000, Kirsch et al., 1995, Zhou and Seeley, 2014). Each of these subprocesses entails complex neural computations that are likely a priori to be vulnerable to the effects of neurodegenerative pathologies. The canonical syndromes of frontotemporal lobar degeneration (FTLD) and Alzheimer's disease (AD) are associated with altered emotional, physiological and behavioural responses to salient sensory signals (Fletcher et al., 2015a, Fletcher et al., 2015b, Fletcher et al., 2015c, Fletcher et al., 2015d, Hoefer et al., 2008, Zhou and Seeley, 2014). These are most strikingly exemplified by the phenotypes of disrupted hedonic valuation and aberrant reward processing that characterise FTLD (Fletcher et al., 2015a, Perry et al., 2014), though AD may also produce abnormalities of sensory salience coding (Fletcher et al., 2015a, Fletcher et al., 2015b, Fletcher et al., 2015c, Fletcher et al., 2015d). Such abnormalities further suggest a physiological substrate for the higher order disturbances of emotional and social cognition that frequently accompany these diseases (Downey et al., 2015, Kumfor and Piguet, 2012, Omar et al., 2011, Warren et al., 2013, Woolley et al., 2015), with implications for biomarker development and management strategies.

The salience of a sensory signal generally depends on attribution of its meaning and this is well illustrated in the often ambiguous realm of sounds. Perceptually similar sound sources can have very different biological implications (compare, for example, the rumble of thunder and the growl of a large predator). Auditory salience cues such as loudness, movement (looming) and affective valence are coded physiologically in pupillary and other autonomic responses (Fletcher et al., 2015c, Fletcher et al., 2015d, Neuhoff, 2001) mediated by distributed cortico-subcortical brain networks (Beissner et al., 2013, Critchley et al., 2000, Mueller-Pfeiffer et al., 2014). In addition to these well recognised examples, auditory semantic ambiguity is also a candidate salience cue: there is a biological imperative to resolve the identity of potentially meaningful sounds, and the ability to do this efficiently and accurately is likely to have conferred survival and reproductive advantages during human evolution. In this context, ‘potentially meaningful’ sounds would include naturally occurring, spectrotemporally complex sounds sharing perceptual characteristics with animal (including conspecific) vocalisations. It might be predicted that the processing of such sounds would engage brain mechanisms for coding salience, particularly under adverse listening conditions where the identity of the sound source is difficult to determine. Coding such ambiguous sounds for salience would direct attentional and behavioural resources to the sound source so that its identity can be determined rapidly with an appropriate behavioural response. From a clinical perspective, diseases of the auditory pathways tend to render sounds more difficult to identify and ‘adverse listening conditions’ might also be produced by brain diseases that degrade central mechanisms of auditory semantic analysis: in this situation, the perceptual features of sounds will be coded more or less accurately but sounds will be ambiguous (and therefore, potentially salient) because the attribution of meaning to auditory percepts is impaired. However, it has not been established whether semantically significant or semantically ambiguous sounds have physiological salience correlates. In particular, the interaction of semantic and salience mechanisms has not been explored in neurodegenerative diseases that that might disrupt these mechanisms differentially.

Here we investigated physiological and neuroanatomical correlates of this putative ‘semantic salience’ response in a cohort of patients representing canonical dementia syndromes (semantic dementia – SD; behavioural variant frontotemporal dementia – bvFTD; progressive nonfluent aphasia – PNFA; typical amnestic AD) relative to healthy older controls. We studied patients representing a range of dementia syndromes in order to assess the extent to which putative salience responses might differentiate or transcend syndromic categories. Semantic deficits are not restricted to a particular syndrome: while SD is the paradigmatic disorder of the human semantic system (Lambon Ralph, Sage, Jones, & Mayberry, 2010), less severe or less consistent auditory and other semantic deficits have been documented in each of the other neurodegenerative syndromes included here (Golden et al., 2015, Goll et al., 2010a, Goll et al., 2011, Hardy et al., 2015, Hsieh et al., 2011). Moreover, these diseases have been shown to have distinct profiles of pupil reactivity to salient sounds (Fletcher et al., 2015c, Fletcher et al., 2015d). We measured pupil responses to sounds that varied in semantic content, constituting two sound conditions: real nonverbal sounds with prior semantic associations and synthetic sounds that lacked any such associations. Pupil responses in these two sound conditions were compared and assessed in relation to nonverbal auditory semantic function in each group. Neuroanatomical correlates were determined using voxel-based morphometry (VBM) of patients' brain MR images. We hypothesised that healthy older individuals would show larger pupil responses to real than synthetic sounds and that the magnitude of this difference would vary inversely with nonverbal auditory semantic function across the patient cohort. In particular, we hypothesised an exaggerated pupil response to real sounds in the SD group, as severely degraded sound identification in these patients would preclude disambiguation of these potentially salient sound sources. We further hypothesised an anatomical correlate of this semantic salience response in anterior temporal cortex previously implicated in auditory semantic analysis (Golden et al., 2015, Goll et al., 2012, Hsieh et al., 2011) and in the central autonomic control network previously implicated in programming physiological salience responses (Critchley et al., 2000, Wang et al., 2014, Wang et al., 2012, Zhou and Seeley, 2014).

## 2 Methods

### 2.1 Participant characteristics

Forty consecutive patients fulfilling consensus criteria for dementia syndromes (bvFTD, *n* = 13; SD, *n* = 11; PNFA, *n* = 6; typical AD, *n* = 10) and 20 healthy older individuals with no history of neurological or psychiatric illness participated. No participants had a clinical history of hearing loss or pupillary disease. All patients with AD (but no other patients) were receiving treatment with acetylcholinesterase inhibitors; four patients were receiving antidepressant medication (two SD, one PNFA, one AD).

In order to assess any effect from peripheral hearing function on experimental performance, screening pure tone audiometry was conducted in each group using a previously described procedure (Golden et al., 2015). A general neuropsychological assessment corroborated the clinical diagnosis for each of the disease groups; all patients had a consistent profile of regional brain atrophy on MRI and none had radiological evidence of significant or strategic vascular damage. Cerebrospinal fluid total tau and beta-amyloid_1-42_ assays (available for 18 patients: seven AD, five bvFTD, four SD, two PNFA) and Florbetapir PET brain imaging (available for six patients: four SD, two PNFA) further supported the clinical diagnoses (AD, CSF total tau: beta-amyloid_1-42_ ratio >1; other groups, CSF total tau: beta-amyloid_1-42_ ratio <1 and Florbetapir-PET negative for amyloid deposition). Genetic screening of the cohort revealed nine patients with a pathogenic mutation (five *C9orf72*; four *MAPT*). Demographic, clinical and general neuropsychological data are summarised in Table 1.

The study was approved by the local institutional ethics committee and all participants gave informed consent in accord with the principles of the Declaration of Helsinki.

### 2.2 Experimental design and methods

#### 2.2.1 Sound stimuli

Nonverbal sounds were derived from publically-available sound libraries (www.freesound.org, www.freesfx.co.uk) and sampled a range of human, animal, environmental and mechanical sounds. In a pilot experiment, 20 healthy younger individuals (median age 28 years (range 23–37), six male) were asked to identify an initial set of 180 sounds: those that were misidentified by two or more (>10%) of the healthy younger pilot control group were excluded in order to ensure all sounds in the main experiment were intrinsically highly familiar and identifiable (final stimulus sets listed in Tables S1 and S2). Audio samples were converted to digital wavefiles sampled at 44.1 kHz, and edited so that all sound stimuli were two seconds in duration (brief sounds such as hiccoughs that tend to be naturally periodic were repeated within this interval) and mean overall intensity (rms value) was fixed across stimuli. In addition, as loudness modulates pupillary dilatation in healthy individuals (Steiner & Barry, 2011) the peak volume of each sound as played through the experimental sound delivery system was measured using a sound level meter and incorporated as a nuisance covariate in subsequent analyses. Ten nonverbal sounds rated as highly identifiable by the pilot healthy control group were selected and spectrally inverted in Matlab^®^ to produce synthetic (‘meaningless’, M−) versions of the sounds that were similarly acoustically complex but lacking the semantic associations of the real (‘meaningful’, M+) sounds. The M+ and M− sounds had matched mean overall intensity; M− sounds had higher measured mean peak loudness than M+ sounds. All sound stimuli were presented via Audio-technica ATH-M50 headphones from a notebook computer at a constant, comfortable listening level (at least 70 dB) in a quiet room.

#### 2.2.2 Pupillometry experiment

Autonomic responses to sounds were assessed using pupillometry. Sounds in the M+ and M− conditions were all presented twice in randomised order, yielding a combined playlist of 40 trials (Table S1). Four additional sounds (two M+, two M−) were presented prior to the playlist proper as familiarisation trials but were excluded from the final analysis.

**Fig. 1:**
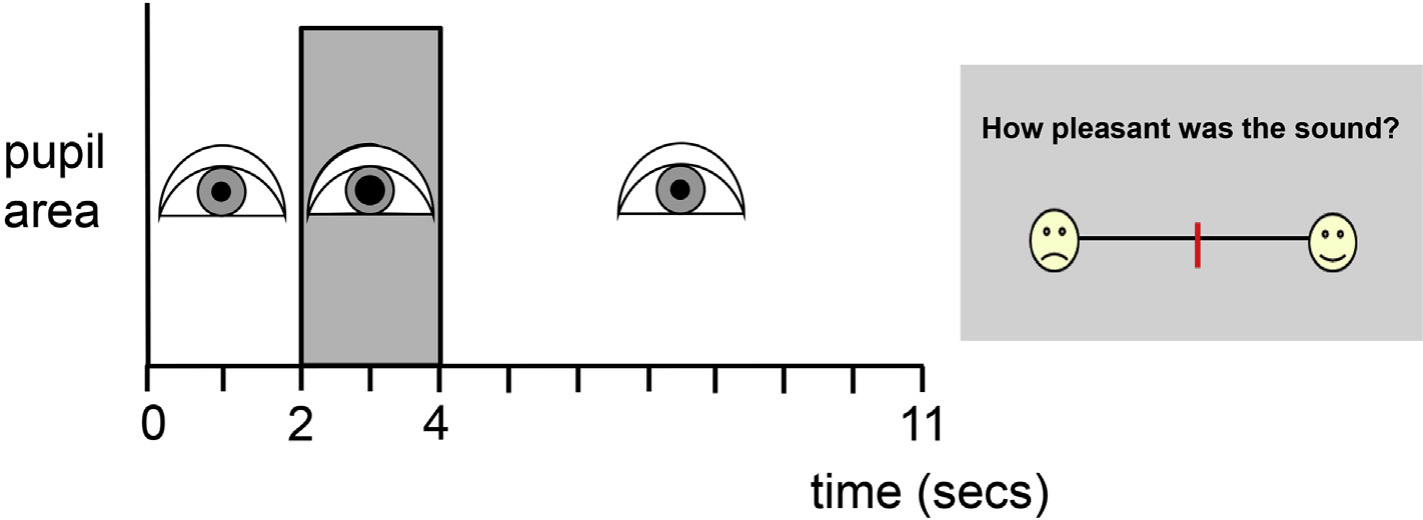
Schematic of trial design in the pupillometry experiment. Area of the right pupil was measured using a headset-mounted infrared camera, while the participant fixated the centre of a monitor screen. Once stable fixation was achieved, a trial was triggered with an initial brief silent interval (2 sec), followed by the sound stimulus (2 sec; shaded rectangle) and a final silent equilibration interval (7 sec). On completion of the recording period, a Likert scale (right) was displayed and the participant was asked to use a wireless mouse to indicate on the line how pleasant and then how alerting they had found the sound; a response triggered the next recording period.

During pupillometry, participants were seated before a computer monitor in a dimly but uniformly illuminated room. The experimental trial design is schematised in Fig. 1. Pupil area was measured from the right pupil using an infra-red camera (Eyelink II; SR Research, Canada) mounted on a headset just below the line of sight while the participant fixated a 1 cm white circle in the centre of the monitor screen. All experiments were run using Eyelink II software. Each experimental trial was triggered once adequate visual fixation was achieved and pupil area was measured (sampling rate 250 Hz) over the entire trial duration. During each trial there was an initial brief silent interval (two seconds), followed by the sound stimulus (two seconds) and a final silent equilibration interval (seven seconds). On completion of the recording period, the participant was asked to rate how pleasant and how alerting they found the sound on modified Likert scales using a wireless mouse cursor, to provide indices of sound affective valence and arousal (no time limit was imposed).

In off-line analysis, baseline pupil size and pupil responses were calculated and artefacts were identified and removed using a customised algorithm in Stata12^®^. This algorithm calculated maximal pupil response as change from baseline pupil area for each trial; baseline values were calculated as the mean value over the initial two second silent interval of the trial. Artefacts were chiefly blinks, easily detected due to their characteristic temporal trajectory; pupil data were discarded for the interval 50 msec prior and 750 msec following an artefact, to allow for completion of an ensuing light reflex (as determined from data collected in the healthy young control pilot group). In a simple regression model, the total proportions of data points removed due to artefacts did not differ significantly (*p* > .05) between sounds or between experimental groups. Maximum pupil area during a given trial was positively correlated with pupil area during the brief silent interval. In order to avoid this potentially confounding influence, the log ratio of maximal pupil area to baseline pupil area was used as the metric of pupil response for each trial (pupil_**max**_).

#### 2.2.3 Auditory semantic classification task

We assessed auditory semantic cognition in the patients and healthy control participants using a novel nonverbal auditory semantic task that did not rely on cross-modal labelling of sounds. Pairs of real (M+) sounds (*n* = 60, incorporating the 10 M+ sounds presented in the pupillometry experiment; see Table S2) were created in which the constituent sounds were associated either with the same sound source (e.g., a goose honking, a goose's wings flapping; 21 trials) or with different sources (e.g., a goose honking, a child yawning; 39 trials). Sound pairs in the ‘same’ and ‘different’ conditions did not differ systematically in the acoustic similarity of their component sounds; sound pair classification therefore relied on a semantic decision based on recognition of the sounds and could not be based on perceptual criteria.

The auditory semantic test was administered separately from the pupillometry session. Sound pairs were presented in randomised order; the sounds comprising each pair were presented serially with a .5 sec inter-sound delay. The task on each trial was to decide whether the paired sounds came from the same source or different sources (‘Are the sounds made by the same kind of thing or different kinds of things?’), and the participant could respond verbally or by pointing to the corresponding written word on a prompt sheet. Participants were given practice trials before commencing the test, to ensure they understood the task and the response criteria; during the test, no feedback about performance was given and no time limits on responses were imposed.

### 2.3 Analysis of behavioural and physiological data

All data were analysed in STATA12.1^®^. Before calculating mean pupil_**max**_ responses for analysis, individual pupil_**max**_ responses to each sound were adjusted for any potential confounding effects of sound loudness, pleasantness and arousal (based on control participant rating data), as these factors are known to drive pupillary reactions (Goldwater, 1972). As individual participants had different numbers of missing data points, the relative effect of these potential confounds might further differ between participants: average effects of loudness and affective factors on pupil_**max**_ were therefore estimated separately using a linear mixed effects model with crossed random effects for participant and sound and this constant value for each sound was added or subtracted from the crude pupil responses. Mean pupil_**max**_ responses to different sound conditions and performance on the sound pair semantic classification task were then compared between participant groups (after adjusting for any demographic differences between groups) and correlations with peripheral hearing function, medication use (whether or not the participant was taking relevant, acetylcholinesterase inhibitor or antidepressant medication), and disease severity metrics (symptom duration and a nonverbal executive measure, reverse visual spatial span) were assessed using linear regression models. The difference in mean pupil_**max**_ responses to the M+ and M− sound conditions was assessed within each group with paired *t*-tests. In order to assess how well the M+ sounds presented in the pupillometry experiment indexed patients' general auditory semantic competence, we performed a separate subanalysis of the semantic classification task to assess just those trials that contained one of the M+ sounds presented in the pupillometry experiment (a subset of 14 semantic classification trials). In all analyses, a threshold *p* < .05 was accepted as the criterion for statistical significance.

### 2.4 Brain image acquisition and analysis

For 26 patients (12 bvFTD, 10 SD, four PNFA), a sagittal 3-D magnetization-prepared rapid-gradient-echo T1-weighted volumetric brain MR sequence (echo time/repetition time/inversion time 2.9/2200/900 msec, dimensions 256 × 256 × 208, voxel size 1.1 × 1.1 × 1.1 mm) was acquired on a Siemens Trio 3T MRI scanner using a 32-channel phased-array head-coil. Pre-processing of brain images was performed using the New Segment (Weiskopf et al., 2011) and DARTEL (Ashburner, 2007) toolboxes of SPM8 (www.fil.ion.ucl.ac.uk/spm) under Matlab 7.0^®^ and following an optimised protocol (Ridgway et al., 2008). Normalisation, segmentation and modulation of grey and white matter images were performed using default parameter settings and grey matter images were smoothed using a 6 mm full width-at-half-maximum Gaussian kernel. A study-specific template mean brain image was created by warping all bias-corrected native space brain images to the final DARTEL template and calculating the average of the warped brain images. In order to adjust for individual differences in global grey matter volumes during subsequent analysis, total intracranial volume (TIV) was calculated for each patient by summing grey matter, white matter and cerebrospinal fluid volumes following segmentation of all three tissue classes.

In the VBM analysis, separate voxel-wise linear regression models were used to assess associations in the combined patient cohort between regional grey matter volume (indexed as voxel intensity) and parameters of interest. Contrasts assessed positive and negative (inverse) correlations between regional grey matter volume and both overall pupil reactivity (individual overall mean pupil_max_ across the sound stimulus set) and differential pupil reactivity to sound semantic content (individual difference in mean pupil_max_ between the meaningful and meaningless sound conditions: M+ > M−). Age, TIV and syndromic group membership were included as covariates of no interest in each model. To help protect against voxel drop-out because of potentially marked local regional atrophy in particular scans, we applied a customised explicit brain mask based on a specified ‘consensus’ voxel threshold intensity criterion (Ridgway et al., 2009), whereby a voxel was included in the analysis if grey matter intensity at that voxel was >.1 in >70% of participants (rather than in all participants, as with the default SPM8 mask). Statistical parametric maps of regional grey matter volume correlating with pupil response parameters of interest were examined at threshold *p* < .05 after family-wise error (FWE) correction for multiple voxel-wise comparisons both at whole brain level and separately within three pre-specified regional volumes of interest. These regional volumes of interest were based on previous neuroanatomical work and encompassed the temporal lobes anterior to Heschl's gyrus (previously implicated in semantic analysis and signalling the behavioural value of sounds and other sensory objects: Goll et al., 2012, Golden et al., 2015, Hsieh et al., 2011), insular cortex (implicated as an ‘autonomic hub’ in salience processing: Critchley et al., 2000, Zhou and Seeley, 2014) and dorsal brainstem including superior colliculi (previously identified as a key integrative site of autonomic effector response: Wang et al., 2012; Wang et al., 2014). Regional volumes were created by manually tracing from the template mean brain image using MRICron^®^.

## 3 Results

### 3.1 General characteristics

The participant groups did not differ in mean age and patient groups did not differ in mean symptom duration; gender distribution and years of education varied between participant groups and were therefore included as nuisance covariates in subsequent analyses. Participant groups did not differ in peripheral hearing performance nor did peripheral hearing performance correlate with pupil response or auditory semantic measures, after exclusion of one outlier patient with bvFTD (performance >2 standard deviations beyond rest of group).

### 3.2 Pupillometric responses

Baseline pupil size did not differ significantly between groups; the PNFA group but not the other groups showed a significant change (increase) in baseline pupil size over the course of the experiment (*p* < .05). Mean pupil_max_ values over the entire sound stimulus set and for the M+ and M− conditions separately did not differ between experimental groups. Over the course of the experiment, healthy individuals showed significant attenuation (*p* < .01) of pupil_max_ responses to M− but preserved responses to M+ sounds; the AD group showed significant (*p* = .03) attenuation of pupil_max_ responses to M+ sounds, while other patient groups showed no significant attenuation of pupil responses over time.

**Fig. 2:**
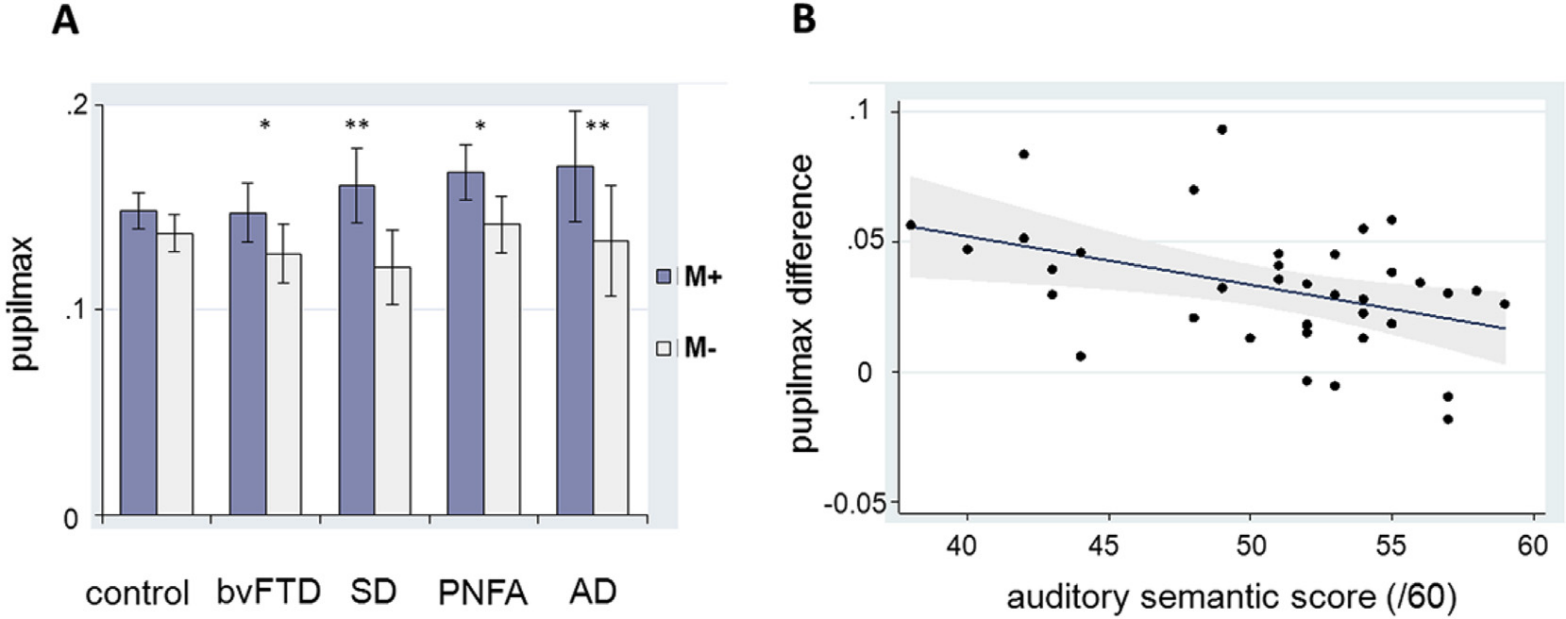
Summary of pupillometric data for sound meaning conditions. A, mean maximal pupil dilatation response (log ratio of maximal pupil area to baseline pupil area, pupil_max_) to real (meaningful, M+) and synthetic (meaningless, M−) sound conditions in each participant group (*significant difference between conditions, **significant difference between conditions and difference significantly greater than healthy controls, *p* < .05; standard error bars shown); B, difference in pupil_max_ between M+ and M− conditions as a function of auditory semantic classification score across the entire patient cohort (linear regression best fit with 95% confidence intervals shown). AD, Alzheimer's disease; bvFTD, behavioural variant frontotemporal dementia; PNFA, progressive nonfluent aphasia; SD, semantic dementia.

M− sounds were rated as less pleasant than M+ sounds by the healthy control group (*p* < .01), but not the patient groups, while M+ and M− sounds did not differ significantly in mean arousal ratings (see Table S1). After adjusting for these factors and for measured peak loudness, the healthy control group showed a non-significant trend (*p* = .09) toward increased mean pupil_max_ responses to M+ compared with M− sounds (see Fig. 2). All patient groups showed significantly greater mean pupil_max_ responses to M+ than to M− sounds (SD, AD, *p* < .01; bvFTD, PNFA, *p* < .05); a post hoc analysis of the small subgroup of patients with pathogenic mutations suggested some heterogeneity within the bvFTD group [significantly greater mean M+ responses than M− responses in *MAPT* mutation cases (*p* < .01), a trend to a significant difference in *C9orf72* mutation cases (*p* = .1) but no significant difference in sporadic bvFTD cases (*p* = .51)]. The increased pupil response to M+ compared M− sounds was significantly larger (*p* < .05) in the SD group and the AD group (though not in other patient groups) than in healthy controls; the magnitude of this differential pupil response did not differ significantly between patient groups. There were no correlations between overall pupil reactivity or the magnitude of the differential pupil_max_ response to M+ versus M− sounds and overall disease severity (as indexed by symptom duration and reverse visual spatial span) or medication use.

### 3.3 Auditory semantic performance

Mean performance for each of the participant groups on the nonverbal auditory semantic classification task is summarised in Table 1. The healthy older control group overall achieved high sub-ceiling scores on this test. Relative to the healthy control group, the PNFA group showed no auditory semantic deficit while each of the other syndromic groups showed impaired performance (all *p* < .01); comparing patient groups, the bvFTD, SD and AD groups performed worse (*p* < .01) than the PNFA group. Auditory semantic performance profiles for the patient groups relative to the healthy control group were similar for the subset of sounds also represented in the M+ condition of the pupillometry experiment. Patients' auditory semantic performance did not correlate with mean overall pupil reactivity or with mean pupil responses to M+ or M− sounds separately. However, auditory semantic performance did show a significant inverse correlation with the magnitude of the difference in mean pupil responses to M+ versus M− sounds, in the combined patient cohort (*r*^2^ = .2; *p* < .01; see Fig. 2) and in each of the patient groups showing an auditory semantic deficit (bvFTD *r*^2^ = .8, *p* < .01; SD *r*^2^ = .6, *p* < .05; AD *r*^2^ = .9, *p* < .01).

### 3.4 Voxel based morphometry associations

**Fig. 3:**
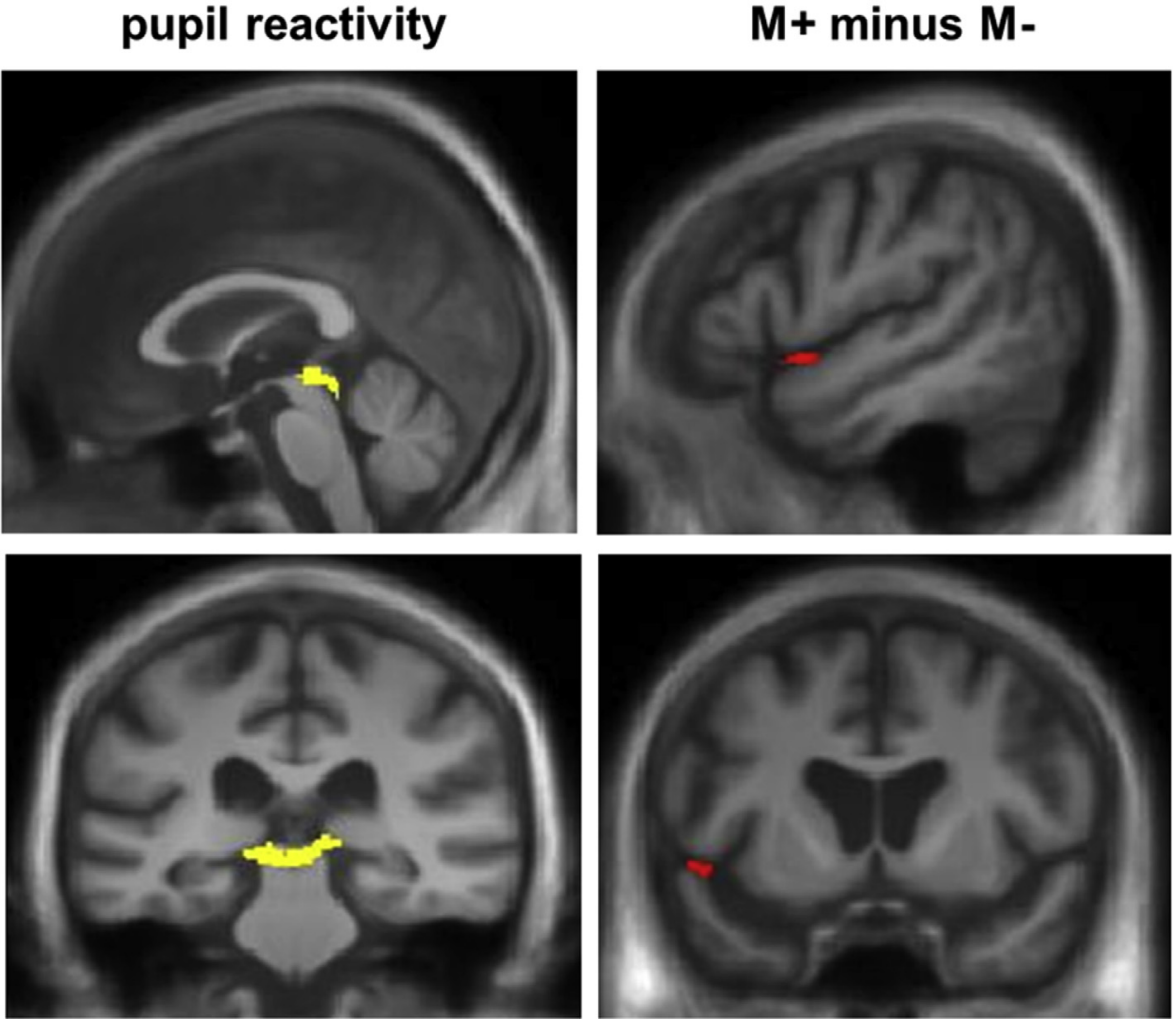
Statistical parametric maps for the combined patient cohort showing regional grey matter significantly positively associated with overall pupil reactivity to sound in superior colliculus (yellow); and inversely associated with the magnitude of the difference in mean maximal pupil dilatation response (pupil_max_) to real (meaningful, M+) over synthetic (meaningless, M−) sounds in left anterior superior temporal cortex (red). All voxel-wise associations shown were significant thresholded at *p*_FWE_<.05 after multiple comparisons correction within anatomical regions of interest (see also Table 2); maps have been rendered on sagittal (above) and coronal (below) sections of a group mean template T1-weighted brain MR image in MNI standard stereotactic space and the left hemisphere is shown on the left in coronal sections.

Grey matter associations of pupil response parameters for the combined patient cohort are summarised in Table 2 and statistical parametric maps are presented in Fig. 3. After FWE correction for multiple voxel-wise comparisons, grey matter associations were not identified at whole brain level but were identified within the pre-specified regional anatomical volumes of interest. Overall pupil reactivity to sound (mean pupil_max_ across the stimulus set) was significantly (*p*_FWE_ < .05) positively correlated with grey matter in the region of the superior colliculus; no grey matter areas showing a significant inverse correlation with overall pupil reactivity were identified. The magnitude of the difference in pupil responses to M+ versus M− sounds was significantly (*p*_FWE_ < .05) inversely correlated with grey matter in left anterior superior temporal cortex; no grey matter areas showing a significant positive correlation with this pupil response difference measure were identified.

## 4 Discussion

Here we have demonstrated that dementia syndromes have different profiles of autonomic responses to real and synthetic nonverbal sounds, after controlling for elementary acoustic and affective factors. This differential autonomic response was present in all patient groups but was largest (and significantly greater than the healthy control response) in patients with SD and AD. Moreover, the magnitude of the differential response was inversely related to auditory nonverbal semantic competence across dementia syndromes but was not related to overall autonomic reactivity, more general disease severity or medication effects. Considered together, these findings suggest that impaired semantic processing of nonverbal sounds confers an enhanced physiological salience signal in these dementia syndromes that is separable from other salience cues such as emotional value and arousal potential.

Little information is available concerning the physiological coding of semantic salience. However, semantic load and in particular, semantic ambiguity along behaviourally relevant dimensions (such as threat) have been shown to modulate cerebral and autonomic responses to both visual and auditory stimuli in the normal brain (Farrow et al., 2012, Werner and Noppeney, 2010). In neurodegenerative diseases, ‘primitive’, behaviourally relevant cues to moving (approaching *vs* withdrawing) sound sources have been shown to modulate autonomic responses and this modulation was enhanced in SD relative to other dementia syndromes (Fletcher, Nicholas, et al., 2015c). Though the evidence remains limited, these previous findings in the healthy brain and in neurodegenerative disease are in line with the present data. If the damaged semantic system cannot identify potentially meaningful sounds, then this unresolved ambiguity may render such sounds behaviourally salient and engage physiological effector mechanisms for salience coding. This autonomic response to auditory semantic salience, though amplified in dementia syndromes, was also evident in attenuated form in the healthy control group here: healthy controls showed a trend toward enhanced pupil responses to real compared with synthetic sounds but (unlike the patient groups) showed habituation of pupil responses to synthetic sounds over the course of the experiment. These data suggest that both highly familiar real sounds and synthetic sounds of the kind presented in the pupillometry experiment are rapidly disambiguated by the normal semantic system (either as meaningful auditory objects or as meaningless sound events).

In this formulation, SD as the paradigmatic disorder of the human semantic system is an important test case: it is noteworthy that here as in previous work (Fletcher, Nicholas, et al., 2015c) patients with SD showed enhanced sensitivity to auditory salience. Compared with other canonical dementia syndromes, patients with SD would be predicted to have the most marked and selective difficulty with disambiguation of meaningful sound sources while retaining relatively intact mechanisms for perceptual coding of sound features and programming autonomic responses (Beissner et al., 2013; Bozeat et al., 2000, Goll et al., 2010a). We do not argue that autonomic responses *per se* are normal in SD: while the present study did not address these processes directly, other work suggests that the coupling of cognitive to autonomic effector processing of sound stimuli is altered in SD as well as in bvFTD and AD (Fletcher et al., 2015c, Fletcher et al., 2015d). However, the processing of complex sounds such as those presented here engages hierarchical and distributed mechanisms both in the healthy brain and in SD (Goll et al., 2010b, Goll et al., 2012), providing candidate neural substrates for relatively intact physiological signalling of auditory salience in patients with dementia. The complex spectrotemporal structure of the stimuli used here would allow the damaged semantic system to encode perceptual features characteristic of real sounds but lacking in the synthetic (spectrally inverted) sounds; the presence of such spectrotemporal features could label natural sounds for further semantic analysis even if identification were not achieved.

A VBM analysis of our combined patient cohort identified neuroanatomical correlates of pupillary responses to sound in a distributed cortico-subcortical network. Midbrain grey matter in the region of the superior colliculus was associated with overall pupillary reactivity to sound. The superior colliculus is involved in orienting responses (Krauzlis et al., 2013, Kustov and Robinson, 1996, Wang et al., 2014, Wang et al., 2012) and in processing potential threat in ambiguous stimuli (Farrow et al., 2012). This region mediates output to the eye, head and neck and arm and shoulder via the thalamus from cortical areas including the frontal eye fields. Stimulation of the superior colliculus results in coordinated head and eye gaze shifts (Freedman, Stanford, & Sparks, 1996) and more recently, it has been shown that stimulation at thresholds below those necessary to evoke saccadic eye movements results in pupillary dilatation in both monkeys and owls (Netser et al., 2010, Wang et al., 2012), suggesting a role for this region in coding salience responses. The present evidence suggests a need for some caution in interpreting the role of superior colliculus in salience coding in neurodegenerative disease. While we did not identify an overall significant impairment of general pupil reactivity across the patient cohort, this autonomic parameter varied between patient groups (for example, the AD group but not the other groups showed significant attenuation of pupil_max_ responses to real sounds over time), suggesting that larger patient cohorts may be required to more fully elucidate the role of this midbrain effector region in salience processing in dementia syndromes.

Enhanced differential pupillary response to auditory semantic salience (real *vs* synthetic sounds) in this patient cohort was associated with atrophy of left anterior temporal cortex. This neuroanatomical association is in line with the inverse association between pupillary salience responses and auditory semantic impairment in the present study and corroborates previous work implicating an anterior temporal lobe network in semantic processing of sounds and other sensory objects (Lambon Ralph et al., 2010, Visser and Lambon Ralph, 2011). The present evidence further suggests that this cortical region is involved in mediating autonomic responses to sound meaning. Although the links between semantic memory and autonomic networks remain poorly defined, current formulations increasingly emphasise the role of distributed cortico-subcortical networks including the anterior temporal lobe in ‘appraising’ the behavioural significance of sensory stimuli and programming appropriate physiological responses (Kafkas and Montaldi, 2012, Zhou and Seeley, 2014). This may be particularly relevant under conditions of perceptual or semantic ambiguity (Farrow et al., 2012, Zekveld et al., 2014): it is therefore plausible that this linkage might be upregulated in the setting of a damaged semantic system.

This study has several limitations that could be addressed in future work. Larger patient cohorts would improve power to detect effects on semantic salience processing and potentially, further stratify dementia syndromes based on both cross-sectional and longitudinal profiles of autonomic reactivity. It would be of particular interest to assess genetic mutation cohorts with defined molecular substrates that are potentially associated with specific auditory salience signatures (Fletcher et al., 2015a, Fletcher et al., 2015b): inclusion of mutation carriers would also allow assessment of the biomarker potential of autonomic indices from earliest clinical disease stages, and might be achieved via multi-centre collaborative studies (Rohrer et al., 2015). Our work leaves open the possibility that other autonomic modalities (such as skin conductance) might show differential sensitivity to semantic impairment, and ideally these modalities would be compared directly. The effects of medications that affect autonomic function should be assessed directly, both in order to calibrate for any confounding impact on endogenous autonomic responses and to determine modulatory effects with therapeutic potential. The linkage between semantic and autonomic processing could be more directly explored using functional neuroimaging; this is particularly pertinent as the interaction of these mechanisms might differ between diseases (Fletcher, Nicholas, et al., 2015d). More fundamentally, the present study suggests a hypothesis concerning the role of auditory semantic ambiguity in triggering physiological salience responses that should be assessed in the healthy brain by manipulating sound stimulus ambiguity and semantic associations systematically.

Taking the above caveats into account, our findings suggest that autonomic responses index semantic impairment across dementia syndromes. The present study should be regarded as preliminary: the sensitivity, specificity and translatability of physiological metrics require further systematic substantiation. Nevertheless, such ‘physiological phenotyping’ of dementia syndromes might be developed as a useful tool in these diseases. The dementias collectively present substantial problems of nosology, diagnosis and disease tracking; these issues are particularly pressing for diseases in the FTLD spectrum for which syndromic boundaries are often difficult to define but robust biomarkers that can be applied across syndromes are lacking. Capturing disease effects near the time of clinical conversion or in the later stages of disease is challenging, as existing neuroanatomical and neuropsychological biomarkers are relatively insensitive; yet accurate disease diagnosis and tracking will be essential to assess the impact of therapeutic interventions in future clinical trials. Studies such as this one could in future be used to guide revised diagnostic criteria for neurodegenerative syndromes, informed by pathophysiological data. Moreover, there is considerable interest in identifying new biomarkers with wider applicability across diseases and disease stages. Autonomic salience signalling warrants further evaluation both as a window on disease neurobiology and as a candidate novel physiological biomarker that could potentially complement or extend the range of conventional cognitive instruments.

## Figures and Tables

**Table 1:** Demographic, clinical and neuropsychological characteristics for participant groups. Maximum total scores are shown (where applicable) after relevant neuropsychological tests; mean (range) data are shown unless otherwise indicated. Statistically significant (*p* < .05) group differences versus the healthy older control group are shown in bold. Other significant differences between groups are indicated by superscripts: a, relative to bvFTD; b, SD; c, PNFA; and d, AD groups. AD, Alzheimer's disease; BPVS, British Picture Vocabulary Scale; bvFTD, behavioural variant frontotemporal dementia; NA, not available; PNFA, progressive nonfluent aphasia; RMT, Recognition Memory Test; SD, semantic dementia; Synonyms, Synonym matching task; VOSP decision, Visual Object and Space Perception battery –object decision task; *general neuropsychological data not available for two patients in the PNFA group and one patient in the AD group; **experimental nonverbal auditory semantic test (see text).

Characteristic	Healthy controls	bvFTD	SD	PNFA*	AD*
**General**					
No.	20	13	11	6	10
Gender (m:f)	10:10	**11:2**	7:4	**1:5**	5:5
Age (y): mean (range)	65.6 (57–71)	65.2 (52–76)	66.5 (53–78)	69.1 (61–77)	68.1 (54–78)
Education (y)	16.9 (12–20)	**14.8 (11–21)**^c^	**14.7 (11–20)**^c^	18 (17–20)	15.2 (12–17)
Symptom duration (y)	NA	7.5 (4–21)	5.2 (3–9)	5.7 (4–10)	5.8 (3–8)
**IQ**					
Verbal	125 (112–137)	**83 (55–10)**^d^	**81 (55–109)**^d^	**93 (70–115)**	**101 (81–129)**
Performance	122 (99–141)	**96 (88–105)**	**109 (88–135)**	**102 (83–121)**	**88 (66–112)**^b^
**Episodic memory**					
RMT words (/50)	48 (42–50)	**34 (25–48)**	**36 (25–47)**	**37 (34–40)**	**32 (30–33)**
RMT faces (/50)	43 (35–50)	**37 (25–41)**	**33 (25–35)**^c^	44 (41–46)	**33 (23–40)**^c^
**Executive functions**					
Stroop word	21 (15–30)	26 (18–39)	26 (14–38)	**53 (43–72)**^a,b^	NA
Stroop inhibition	51 (35–70)	**100 (48–180)**	**81 (36–136)**	**122 (75–180)**	NA
Digit span reverse (max)	5.5 (3–7)	4.5 (3–6)	5.6 (3–8)	**4.2 (3–7)**	5.3 (3–8)
**Visuoperceptual functions**					
VOSP decision (/20)	19 (16–20)	**17 (13–20)**	**17 (14–20)**	18 (16–19)	**16 (12–18)**^c^
**Semantic processing**					
BPVS (/150)	148 (146–150)	**132 (102–147)**	**97 (41–147)**^a,c,d^	141 (131–145)	140 (120–148)
Synonyms (50)	49 (48–50)	**37 (20–47)**	**34 (20–49)**	41 (31–48)	44 (41–46)
Sound classification (60)**	58 (51–60)	**48 (38–57)**^c^	**50 (40–57)**^c^	56 (54–59)	**51 (43–55)**^c^

**Table 2:** Grey matter regions associated with key experimental parameters in the voxel-based morphometry analysis of the combined patient cohort are shown, together with coordinates of local maxima in MNI standard stereotactic space with associated Z-scores, and cluster sizes (number of voxels). Maxima shown were significant at threshold *p*_FWE_<.05 corrected for multiple comparisons within anatomical small volume of interest, based on prior hypotheses (see text).* individual overall mean pupil_max_ over the sound stimulus set; M+ meaningful (real) sounds; M− meaningless (synthetic) sounds.

Parameter	Contrast	Region	Local max (mm)			*Z*-score	Cluster (voxels)
*x*	*y*	*z*
Overall pupil reactivity*	Positive correlation	Superior colliculus	−12	−27	−6	4.12	499
Difference in mean pupil responses M+ > M−	Inverse correlation	Temporal pole	−51	9	−8	4.29	74
