# Supplementary material for: A physiological signature of sound meaning in dementia
Source: Cortex. 2016 Apr;77:13–23. doi: 10.1016/j.cortex.2016.01.007 (PMC4819950; doi:10.1016/j.cortex.2016.01.007)
Supplement: Supplementary file 1 [file mmc1.docx]

**Supplementary material:**

| **Sound stimuli** | **Healthy controls** | | **bvFTD** | | **SD** | | **PNFA** | | **AD** | |
| --- | --- | --- | --- | --- | --- | --- | --- | --- | --- | --- |
| **Real sounds (M+)** | pleas | alert | pleas | alert | pleas | alert | pleas | alert | pleas | alert |
| Bees humming | 3 | 7.2 | 3.3 | 7.2 | 3.1 | 6.8 | 3 | 6.1 | 3.4 | 7.2 |
| Brushing teeth | 4.4 | 6 | 3.9 | 7.6 | 4.7 | 6.5 | 3.6 | 5.8 | 4.5 | 6.2 |
| Car horn | 4 | 7.2 | 4.1 | 7.7 | 5.4 | 7.6 | 3.5 | 6 | 3.5 | 7.5 |
| Car engine running | 4.1 | 6.4 | 4 | 7.3 | 5 | 6.1 | 4.1 | 5.6 | 3.9 | 6.9 |
| Stream babbling | 5.5 | 6 | 5.4 | 6.3 | 5.2 | 6.3 | 4.9 | 5.5 | 5.5 | 5.7 |
| Telephone receiver replace | 4 | 6.3 | 4.2 | 6.7 | 3.2 | 6.8 | 3.1 | 6 | 3.6 | 6.8 |
| Telephone ringing | 4 | 7.5 | 4.2 | 7.7 | 5.1 | 7.4 | 3.6 | 5.8 | 4.4 | 7.2 |
| Thunder | 4.3 | 6.9 | 4.1 | 7.1 | 4.8 | 7.2 | 3.5 | 6.5 | 4.2 | 6.8 |
| Train horn | 4.3 | 7.5 | 4.6 | 6.9 | 5.5 | 6.9 | 3.8 | 6.3 | 4.4 | 6.7 |
| Waves lapping | 4.4 | 6.7 | 4.1 | 7.3 | 4.3 | 7.3 | 3.7 | 6.3 | 4.7 | 7 |
| *mean* | 4.2 | 6.8 | 4.2 | 7.2 | 4.6 | 6.9 | 3.7 | 6 | 4.2 | 6.8 |
| **Synthetic sounds (M-)** |  |  |  |  |  |  |  |  |  |  |
| Synth[Bees humming] | 3.5 | 7.1 | 3.7 | 7.8 | 4.1 | 6.7 | 3.1 | 5.3 | 3.3 | 7.2 |
| Synth[Brushing teeth] | 3.4 | 7.1 | 3.8 | 7 | 3.7 | 7.8 | 3.5 | 6.1 | 3.6 | 7.2 |
| Synth[Car horn] | 4.1 | 6.8 | 4.1 | 6.8 | 3.9 | 6.5 | 3.2 | 6.3 | 4.2 | 6.6 |
| Synth[Car engine] | 3.8 | 6.8 | 3.8 | 7.1 | 4.6 | 7.3 | 3.2 | 6.1 | 4 | 7.1 |
| Synth[Stream babbling] | 3.6 | 7 | 3.7 | 7.3 | 3.9 | 7.4 | 3.1 | 5.9 | 3.7 | 7.2 |
| Synth[Telephone receiver] | 3.4 | 7.1 | 3.9 | 7.4 | 4 | 6.8 | 3.3 | 5.4 | 3.3 | 7.4 |
| Synth[Telephone ringing] | 3.5 | 6.9 | 3.6 | 7.1 | 4.3 | 6.5 | 2.9 | 6 | 3.8 | 7.2 |
| Synth[Thunder] | 3.8 | 6.8 | 4.1 | 7.2 | 3.9 | 7.3 | 3.4 | 6.1 | 3.9 | 6.9 |
| Synth[Train horn] | 3.9 | 6.7 | 3.7 | 7.4 | 4 | 6.3 | 3.3 | 6.2 | 4.4 | 7.2 |
| Synth[Waves lapping] | 3.6 | 7 | 3.8 | 7.7 | 4.2 | 7.3 | 3 | 6.1 | 3.4 | 7.3 |
| *mean* | 3.7 | 6.9 | 3.8 | 7.3 | 4.1 | 7 | 3.2 | 5.9 | 3.8 | 7.1 |

**Fletcher PD et al., A physiological signature of sound meaning in dementia**

**Table S1**. Real (‘meaningful’, M+) sounds and synthetic (‘meaningless’, M-) sounds presented in the stimulus playlist for the pupillometry experiment; for each sound, mean affective valence (pleasantness, pleas) and arousal (alerting, alert) ratings based on Likert scales (1, very unpleasant / not at all alerting – 10, very pleasant / alerting) in each expermental group are shown. Sounds were each presented twice in randomised order during the experiment. AD, Alzheimer’s disease; bvFTD, behavioural variant frontotemporal dementia; PNFA, progressive nonfluent aphasia; SD, semantic dementia.

| **Pair no.** | **‘different’ sound pairs** | |
| --- | --- | --- |
| 1 | baby cooing | stream babbling |
| 2 | baby laughing | waves lapping |
| 3 | brushing teeth | train horn |
| 4 | car alarm disarmed | shovel on metal |
| 5 | car horn | grandfather clock ticking |
| 6 | car skidding | man snoring |
| 7 | cat whining | puppy yelping |
| 8 | child yawning | geese honking |
| 9 | clapping hands | dog barking |
| 10 | clock ticking | infant sobbing |
| 11 | cuckoo clock chime | person breaking wind |
| 12 | dog growling | woman coughing |
| 13 | dog lapping water | child hiccoughing |
| 14 | fizzy drink can opened | coin dropped on table |
| 15 | hen clucking | man sighing |
| 16 | horse trotting | walking on gravel |
| 17 | horse whinnying | woman yawning |
| 18 | infant sneezing | car engine running |
| 19 | infant wailing | man wheezing |
| 20 | man shouting in pain | car horn |
| 21 | man sobbing | bees humming |
| 22 | man vomiting | clock alarm bell |
| 23 | mosquito | woman screaming |
| 24 | paper rustling | woman clearing throat |
| 25 | paper tearing | child hiccoughing |
| 26 | pigeon cooing | fingers clicking |
| 27 | pigeon wings flapping | wind soughing |
| 28 | pigeon wings flapping | person chewing |
| 29 | shovel digging gravel | car window winder |
| 30 | telephone dial tone | man clearing throat |
| 31 | telephone engaged tone | cat hissing |
| 32 | telephone receiver replace | woman coughing |
| 33 | telephone ringing | person breathing |
| 34 | thunder | car engine starting |
| 35 | train travelling on tracks | infant coughing |
| 36 | waves crashing | woman giggling |
| 37 | woman crying | car crash sounds |
| 38 | woman humming | water running |
|  | **‘same’ sound pairs** | |
| 39 | man shouting in pain | man vomiting |
| 40 | baby cooing | baby laughing |
| 41 | baby coughing | man snoring |
| 42 | car crash sounds | car horn |
| 43 | car horn | car skidding |
| 44 | child yawning | child hiccoughing |
| 45 | clock ticking | clock alarm bell |
| 46 | cockerel crowing | hen clucking |
| 47 | geese honking | goose wings flapping |
| 48 | grandfather clock chime | grandfather clock ticking |
| 49 | horse whinnying | horse trotting |
| 50 | infant wailing | infant sobbing |
| 51 | man clearing throat | man sighing |
| 52 | man sobbing | man breaking wind |
| 53 | pigeon wings flapping | pigeon cooing |
| 54 | stream babbling | waves lapping |
| 55 | telephone ringing | telephone dial tone |
| 56 | train travelling on tracks | train horn |
| 57 | water running | waves crashing |
| 58 | woman clearing throat | woman yawning |
| 59 | woman crying | woman screaming |
| 60 | woman giggling | woman humming |

**Table S2**. Sound pair stimuli presented in the nonverbal auditory semantic classification test; pairs are numbered here for ease of reference but were presented in randomised order across conditions. ‘Different’ condition, different sound sources; ‘same’ condition, same sound source.
